# Electrospray Deposition of ZnO Thin Films and Its Application to Gas Sensors

**DOI:** 10.3390/mi9020066

**Published:** 2018-02-02

**Authors:** Wenwang Li, Jinghua Lin, Xiang Wang, Jiaxin Jiang, Shumin Guo, Gaofeng Zheng

**Affiliations:** 1 School of Mechanical and Automotive Engineering, Xiamen University of Technology, Xiamen 361024, China; xmlww@xmut.edu.cn (W.L.); ljh@stu.xmut.edu.cn (J.L.); 2 Department of Instrumental and Electrical Engineering, Xiamen University, Xiamen 361005, China; jiangjx@xmu.edu.cn; 3 Xiamen Key Laboratory of Optoelectronic Transducer Technology, Xiamen 361005, China; 4 Fujian Key Laboratory of Universities and Colleges for Transducer Technology, Xiamen 361005, China; 5 School of Mathematical Sciences, Xiamen University, Xiamen 361005, China; shumin_guo@xmu.edu.cn

**Keywords:** electrospray, ZnO, gas sensor, semiconductor

## Abstract

Electrospray is a simple and cost-effective method to fabricate micro-structured thin films. This work investigates the electrospray process of ZnO patterns. The effects of experimental parameters on jet characteristics and electrosprayed patterns are studied. The length of stable jets increases with increasing applied voltage and flow rate, and decreases with increasing nozzle-to-substrate distance, while electrospray angles exhibit an opposite trend with respect to the stable jet lengths. The diameter of electrosprayed particles decreases with increasing applied voltage, and increases with flow rate. Furthermore, an alcohol gas sensor is presented. The ZnAc is calcined into ZnO, which reveals good repeatability and stability of response in target gas. The sensing response, defined as the resistance ratio of *R*_0_*/R_g_*, where *R*_0_ and *R_g_* are resistance of ZnO in air and alcohol gas, increases with the concentration of alcohol vapors and electrospray deposition time.

## 1. Introduction

Inkjet printing [1] has attracted attention for the fabrication of micro/nano-functional structures as it is a non-contact technology that does not require etching and exposure processing. It allows complex functional patterns to be printed precisely on flat and three-dimensional substrates. Different printing and deposition techniques have been developed. Among these, electrospray deposition [2] is of particular interest as it represents an alternative to conventional deposition and photolithographic patterning techniques for various functional structures. Electrospray is an emerging technology that utilizes electric fields to realize liquid jet ejection to fabricate micro-/nano-scale droplets, particles, and thin films [3]. The electrostatic field can cause the liquid jets to be broken into smaller particles with diameters between 10 and 500 nm by charge repulsion. Electrospray involves the use of simple equipment and has excellent material compatibility, which allows the fabrication of a diverse range of materials, including polymers [4,5], semiconductors [6], and bio-particles [7].

Zinc oxide (ZnO) [8] is a semiconductor with a wide band gap and a high electron mobility. ZnO nanostructures exhibit desirable physical and chemical properties for use in a variety of applications, including electronic devices [9], sensors [10,11] and optoelectronic devices [12]. There are a number of different methods used to fabricate ZnO films [13,14], including ultrasonic spraying, thermal evaporation, and chemical vapor deposition, etc. Among these methods, electrospray is a simple and cost-effective way to fabricate microstructured thin films. In this paper, we report the use of electrospray to deposit ZnO patterns. The effects of experimental parameters on electrospray jet characteristics and electrosprayed patterns are studied. Additionally, a gas sensor is fabricated and the sensing response is demonstrated.

## 2. Materials and Methods

Zinc acetate dihydrate (ZnAc) and glycerol are used to prepare the solution for electrospray. Briefly, the ZnAc is added to the glycerol, and the concentration of ZnAc is 4 wt %. The ZnAc/glycerol solution is stirred at room temperature for 24 h to form a homogeneous solution.

**Figure 1:**
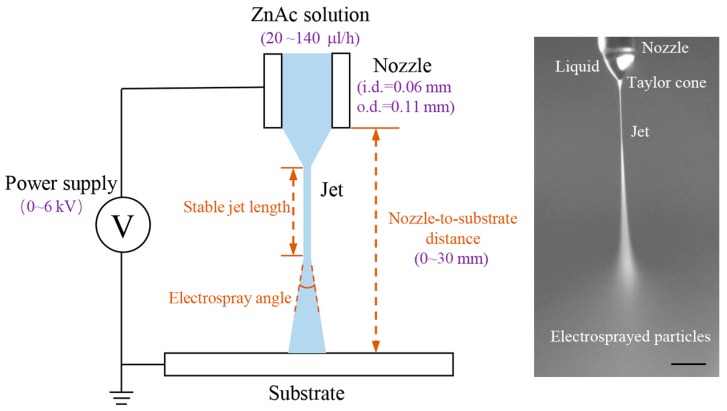
Schematic of the experimental setup. The optical image inset illustrates the electrospray process of ZnAc solution; scale bar: 0.1 mm.

The schematic of the experimental setup is shown in Figure 1. The ZnAc/glycerol solution is loaded into a syringe equipped with a capillary steel nozzle with an inner diameter of 0.06 mm and an outer diameter of 0.11 mm. A precise syringe pump (Harvard 11 Pico Plus, Harvard Apparatus, Cambridge, MA, USA) feeds the syringe at a controllable flow rate. A high-voltage power supply (DW-P403-1AC, Tianjin Dongwen High Voltage Power Supply Plant, Tianjin, China) with the anode connected to the nozzle and the cathode connected to a grounded glass substrate is used to generate the electric field for electrospray. The morphology of electrosprayed patterns is examined using an optical microscope.

The ZnAc/glycerol solution is served as a precursor. To generate the ZnO thin film, the electrosprayed ZnAc pattern is placed on a heating plate (C-MAG HS7, IKA^®^-Werke GmbH & Co. KGStaufen, Breisgau, Germany) to conduct a calcination process. This calcination process is conducted at a temperature of 773 K in air. The heating rate is set at 10 K/min and the heating time is 1 h. After the calcination, the ZnAc/glycerol is decomposed and oxidized to obtain ZnO.

The electrospray process is observed and captured by a CCD camera (UI-1250SE, IDS Imaging Development Systems GmbH, Obersulm, Germany) equipped with a zoom lens. The electrosprayed patterns are observed by an optical microscope (FS-70, Mitutoyo Co., Kawasaki, Japan). The stable jet length, electrospray angle and particle diameter are measured with the assistance of an image processing and analysis software (ImageJ, National Institutes of Health, Bethesda, MD, USA).

## 3. Results and Discussion

In the typical electrospray apparatus, a high voltage is applied to a fluid supplied though a capillary nozzle. The liquid drop on the nozzle tip subjects an electrostatic force on the interface as a result of electric field generated between the nozzle and collector. This electrostatic force opposes the surface tension force and deforms the liquid into a conical shape, called Taylor cone [15]. If the electric field applied is large enough, the electric field force will overcome the surface tension force, results a liquid jet ejected through the apex of the Taylor cone to the collector. The liquid jet travels a straight path and then breaks into small charged liquid droplets/particles which are radially dispersed due to the Coulomb repulsion [16].

**Figure 2:**
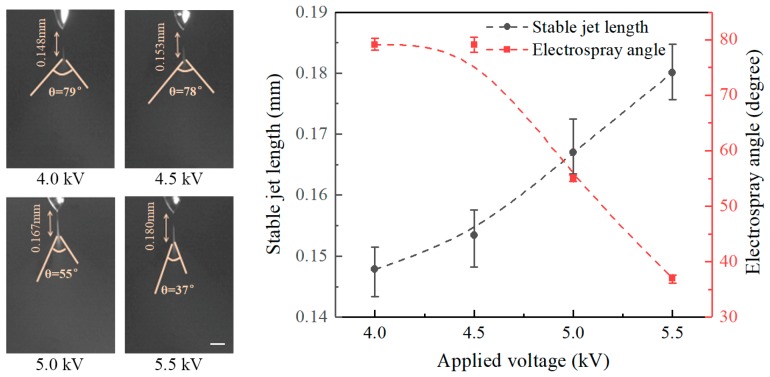
Effect of applied voltage on the stable jet length and electrospray angle. The nozzle-to-substrate distance is 20 mm and the flow rate is 20 μL/h. Scale bar: 0.1 mm.

Firstly, the influence of experimental parameters on the electrospray process is investigated. Figure 2 illustrates the effect of the applied voltage on the length of the stable jets and on the electrospray angles at a nozzle-to-substrate distance of 20 mm and a flow rate of 20 μL/h. The electrospray process maintains a stable cone-jet ejection when the applied voltage is above 3 kV. The length of stable jets increases with increasing applied voltage and the electrospray angle decreases when the applied voltage is enlarged. The stable jet lengths at applied voltages of 4, 4.5, 5, and 5.5 kV are 0.148, 0.153, 0.167, and 0.180 mm, and the corresponding electrospray angles are 79°, 78°, 55°, and 37°, respectively. Theoretically, the axial electric field intensity increases with increasing applied voltage. Under such conditions the liquid experiences larger electric field forces. In this case, both axial acceleration, as well as axial velocity increase, resulting in longer stable jets and smaller electrospray angles. Although the radial electric field also increases with the applied voltage, the axial field dominates under such experimental conditions.

**Figure 3:**
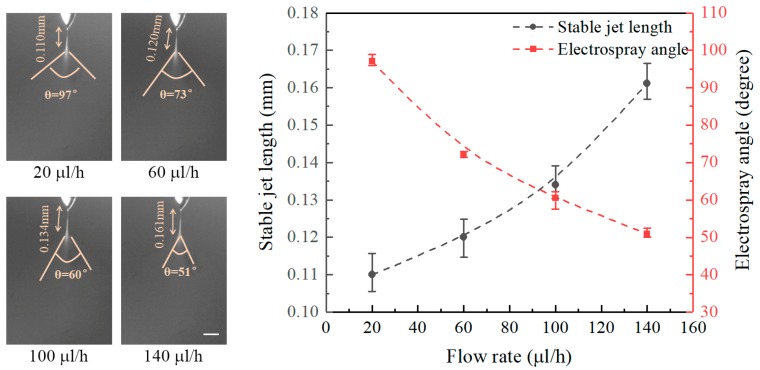
Effect of flow rate on the stable jet length and electrospray angle. The nozzle-to-substrate distance is 20 mm and the applied voltage is 6 kV. Scale bar: 0.1 mm.

Figure 3 shows the effect of flow rate on the stable jet lengths and on the electrospray angles at a fixed nozzle-to-substrate distance of 20 mm and an applied voltage of 6 kV. It can be seen that the stable jet lengths increase with increasing flow rate, while the electrospray angles decrease with the flow rate. The stable jet lengths at flow rates of 20, 60, 100, and 140 μL/h are 0.110, 0.120, 0.134, and 0.161 mm, and the corresponding electrospray angles are 97°, 73°, 60°, and 51°, respectively. Increasing flow rates accelerate the liquid ejection, thus increasing the stable jet lengths and decreasing the electrospray angles.

**Figure 4:**
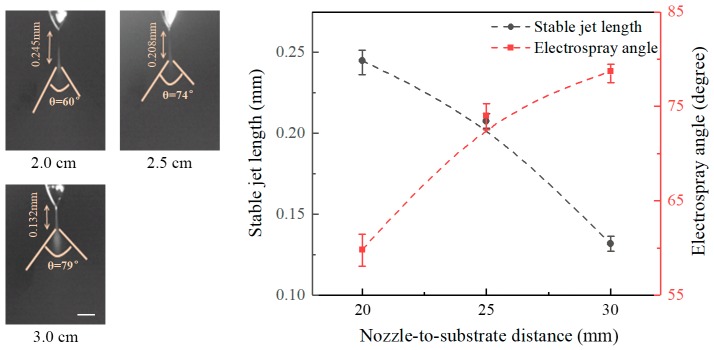
Effect of nozzle-to-substrate distance on the stable jet length and electrospray angle. The applied voltage is 6 kV and the flow rate is 20 μL/h. Scale bar: 0.1 mm.

Figure 4 shows the effect of nozzle-to-substrate distance on the stable jet length and the electrospray angle at an applied voltage of 6 kV and a flow rate of 20 μL/h. It reveals that the length of stable jets decreases with increasing nozzle-to-substrate distance, while the electrospray angle increases with the nozzle-to-substrate distance. Stable jet lengths at nozzle-to-substrate distances of 20, 25, and 30 mm are 0.245, 0.208, and 0.132 mm, and the corresponding electrospray angles are 60°, 74°, and 79°, respectively. Increasing the nozzle-to-substrate distances reduces the electric field intensity, the force acting on the liquid is decreased such that the nozzle-to-substrate distance is shortened, and the electrospray angle is enlarged.

**Figure 5:**
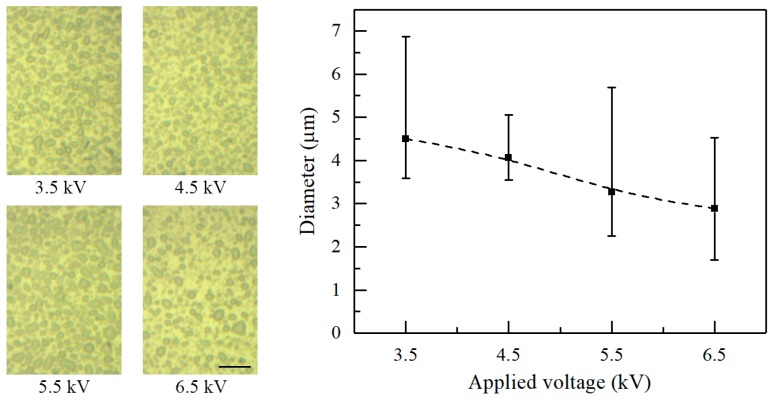
Effect of applied voltage on the diameter of the electrosprayed particles. The nozzle-to-substrate distance and the flow rate are 10 mm and 20 μL/h, respectively. The optical photographs inset are the electrosprayed particles. Scale bar: 20 μm.

The experimental parameters also impact the morphology of the deposited patterns. Figure 5 shows the morphology of deposited patterns under various applied voltages. The nozzle-to-substrate distance and the flow rate are 10 mm and 20 μL/h, respectively. The average diameter of the electrosprayed particles decreases with increasing applied voltage. The average diameter is 4.51, 4.07, 3.27, and 2.88 μm when the applied voltage is 3.5, 4.5, 5.5, and 6.5 kV, respectively. Actually, increasing the applied voltages increases the electric field intensity and the charge density on the solution surface. Increasing the surface charge density increases the Coulomb repulsion within the ejected liquid, which contributes to the atomization of the charged jets.

**Figure 6:**
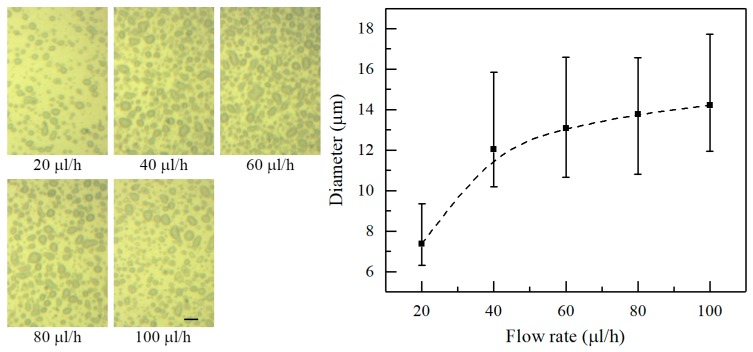
Effect of the flow rate on the diameter of electrosprayed particles. The nozzle-to-substrate distance and the applied voltage are 10 mm and 2.5 kV, respectively. The optical photographs inset are the electrosprayed particles. Scale bar: 20 μm.

Figure 6 shows the morphology of the deposited patterns under various flow rates at a nozzle-to-substrate distance of 10 mm and an applied voltage of 2.5 kV, indicating an increase in the average diameter of electrosprayed particles with increasing flow rate. The average diameter is 7.42, 12.09, 13.10, 13.78, and 14.24 μm when the flow rate is 20, 40, 60, 80, and 100 μL/h, respectively. As mentioned above, large flow rates favor fast liquid ejection. The charged jet do not have enough time to atomize under the short nozzle-to-substrate distance, resulting in larger electrosprayed particles.

**Figure 7:**
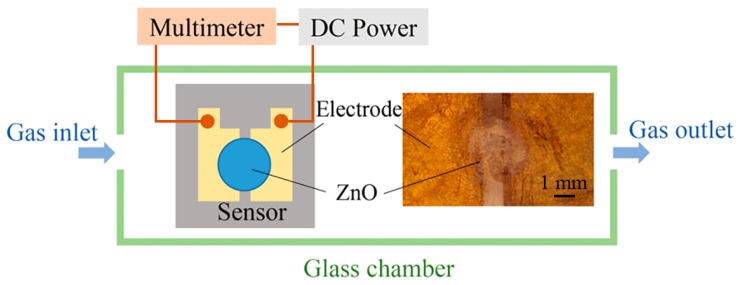
Diagrammatic sketch of the prepared gas sensor and the measuring system. The gap between the two electrodes is about 1 mm. The diameter of ZnO deposition area is in the range of 1.5–2.5 mm.

To demonstrate the feasibility of electrospray processes in the field of sensor manufacturing, an alcohol gas sensor using ZnO patterns as a sensitive layer is fabricated on a silicon substrate. This substrate is pre-processed to form two electrodes with a gap of 1 mm. The ZnAc solution is electrosprayed over the gap between the two Au electrodes, and then calcined to form ZnO structures. The applied voltage, flow rate, and nozzle-to-substrate distance are 1.5 kV, 50 μL/h, and 0.5 mm, respectively. A diagrammatic sketch of the prepared gas sensor and the measuring system is shown in Figure 7. The two electrodes of the alcohol gas sensor are connected to the anode and cathode of the DC voltage supply (GPC3060D, Gwinstek, New Taipei City, Taiwan), and the current passing through the sensor is measured by a digital multimeter (34410A, Agilent Technologies, Santa Clara, CA, USA). The changes in the ZnO resistance in alcohol vapor and in air are evaluated to calculate the sensing response. The sensing response is defined as *R*_0_*/R_g_*, where *R*_0_ and *R_g_* are the resistances of ZnO in air and in alcohol vapor, respectively.

**Figure 8:**
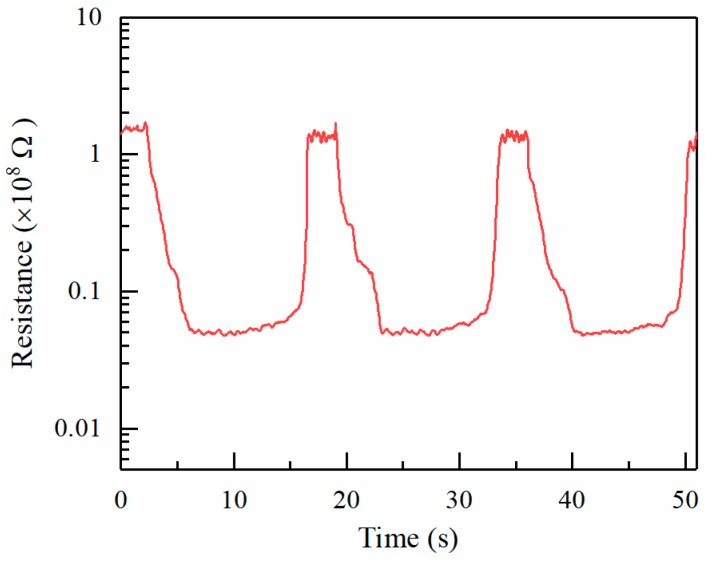
Response of the gas sensor towards repeated exposures to the alcohol vapor. The deposition time for ZnO is 15 min. The concentration of alcohol is 150 ppm.

**Figure 9:**
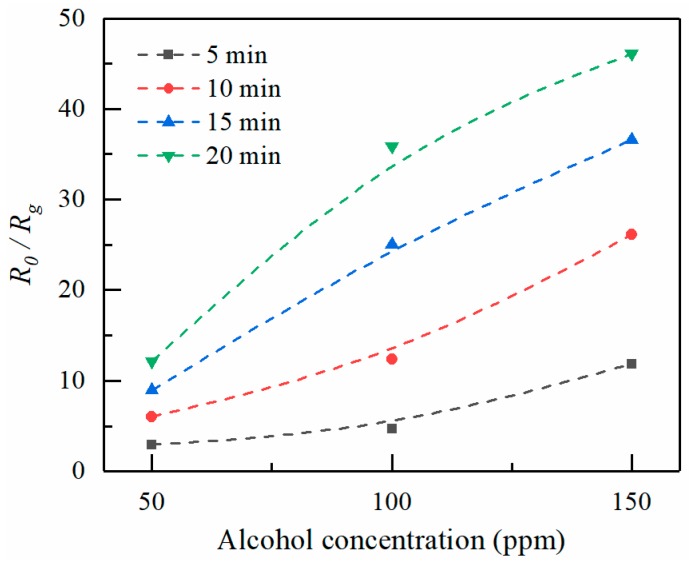
Sensing response of ZnO thin films formed at increasingly longer electrospray times and exposed to increasingly large alcohol concentrations.

When the ZnO is exposed to alcohol vapor, the alcohol molecules adsorb on the ZnO surface, resulting in a decreased resistance. Figure 8 shows the sensing response of ZnO towards repeated exposures to 150 ppm alcohol vapor. The deposition time for electrospray is 15 min. The resistance of the sensitive material in air and alcohol are about 1.55 × 10^8^ Ω and 0.43 × 10^7^ Ω, respectively. The sensing response *R*_0_*/R_g_* is about 36. Moreover, it reveals a good repeatability and stability of response in the target gas. Figure 9 shows the sensing response of ZnO thin films formed at increasingly larger electrospray times as a function of alcohol concentration. Higher vapor concentrations cause more molecules to absorb on the ZnO surface, thus resulting in a resistance decrease. Thus, *R*_0_*/R_g_* increases with increasing alcohol concentration. On the other hand, *R*_0_*/R_g_* increases with increasing electrospray deposition time. Longer ejection periods lead to larger amounts of sensor elements, as the thickness of the ZnO deposition increases with increasing electrospray time. The diameter of ZnO deposition area is in the range about 1.5–2.5 mm. The average thicknesses for various deposition times of 5, 10, 15, and 20 min are estimated to be 100, 130, 180, and 200 nm, respectively.

## 4. Conclusions

This study investigates the effect of experimental parameters on the electrospray process of ZnAc. We have shown that the stable jet length increases with increasing applied voltage and flow rate, while it decreases with increasing nozzle-to-nozzle substrate distance. The corresponding electrospray angles show an opposite in contrast to the stable jet lengths. The diameter of electrosprayed particles decreases with increasing applied voltage, and increases with the flow rate. Furthermore, an alcohol gas sensor has been demonstrated by electrospray and calcining processes. The ZnAc is calcined into ZnO, which reveals good repeatability and stability of response in the target gas. The sensing response, defined as the resistance ratio of *R*_0_*/R_g_*_,_ increases with the concentration of alcohol vapors and the deposition time of electrospray.
